# Cystatin C as a Marker of Kidney Function in Children

**DOI:** 10.3390/biom14080938

**Published:** 2024-08-02

**Authors:** Megan Skidmore, Sebastian Spencer, Robert Desborough, David Kent, Sunil Bhandari

**Affiliations:** 1 Faculty of Medicine, Hull York Medical School, Hull HU6 7RU, UK; 2 School of Medical Sciences, University of Hull, Hull HU6 7RX, UK; 3 Academic Renal Research, Hull University Teaching Hospitals NHS Trust, Hull HU3 2JZ, UK; 4 Paediatric Department, Clarendon Wing, Leeds General Infirmary, Leeds Teaching Hospitals NHS Trust, Leeds LS1 3EX, UK

**Keywords:** acute kidney injury, biomarker, chronic kidney disease, estimated glomerular filtrate rate, paediatric, urinary tract malformations

## Abstract

This review examines the reliability of cystatin C as a biomarker for kidney function in paediatric populations. Chronic kidney disease (CKD) affects a significant number of children globally, leading to severe health complications such as anaemia, hypertension, and growth disorders. Traditionally, kidney function has been assessed using the estimated glomerular filtration rate derived from serum creatinine, though this method is flawed due to variability in muscle mass, age, gender, and diet. Cystatin C offers an alternative as it is less influenced by these factors. Evidence from various studies indicates that cystatin C provides a more accurate assessment of kidney function, especially in neonates and children with urinary tract malformations. Additionally, it is more reliable in early detection of acute kidney injury in paediatric intensive care units. Despite its potential, cystatin C is not yet widely adopted in clinical guidelines, primarily due to a lack of large-scale paediatric studies. Nonetheless, existing research supports its utility in providing a consistent and precise measure of kidney function across different paediatric age groups, suggesting that it could enhance early diagnosis and management of CKD in children if more extensive validation studies are conducted.

## 1. Introduction

The kidneys perform multiple functions, including excretion of waste products, regulation of electrolytes and fluids, metabolism of medications, and several endocrine roles. Chronic kidney disease (CKD) is defined as abnormalities of kidney structure or function present for greater than 3 months, with implications for health. This equates to a glomerular filtration rate (GFR) of less than 60 mL/min/1.73 m^2^, or the presence of at least one of proteinuria; albuminuria; abnormal urinary sediment, such as haematuria; electrolyte abnormalities due to tubular disorders; abnormalities detected by histology or imaging; genetic kidney disease; or a history of kidney transplantation. This definition does encapsulate paediatric considerations (birth-18 years), with some exceptions: a duration over 3 months is not applicable to infants ≤ 3 months of age; there should be age-appropriate adaptations in those under 2 years to the GFR criteria of <60 mL/min/1.73 m^2^; and urinary total proteinuria and albuminuria should be substituted for albuminuria ≥ 30 mg/24 h and all electrolyte abnormalities according to age-normative values [1]. In infants under 2 years, normal GFR values are lower than 60 mL/min/1.73 m^2^, making it difficult to define CKD. A decrease of one standard deviation (SD) is defined as mild, two SD is moderate, and three SD severe. The normal reference values defined by Schwartz et al. are used in this situation [2,3].

It is estimated that 9% to 13% of the adult population worldwide has CKD [4], with this burden leading to anaemia, increased risk of cardiovascular disease, hyperparathyroidism, and renal osteodystrophy as it progresses [5]. Kidney disease in children is relatively uncommon. However, it can have significant and long-term implications, including reduced life expectancy, hypertension, anaemia, and mineral bone disorders, all of which may affect growth and maturation [6]. There are also further ramifications on the psychosocial aspects of child development, such as reduced education opportunities, a higher incidence of behavioural and relationship problems, and issues with self-esteem [7].

CKD rates in children represents a significant cause of morbidity globally, with a prevalence of 15 to 74.7 cases/million children [8]. In the UK, in 2021, 110 children under 16 (9/million) received kidney replacement therapy (dialysis or kidney transplant) for kidney failure. This is in addition to the 842 children below 16 years already on these treatments. Conditions affecting the structure of the kidney or urinary tract were the most common causes of starting treatment, with familial and inherited conditions being the most common cause of kidney failure overall, with over half (58%) of patients being male and of a white ethnic background (67%) [9].

Haemolytic uraemic syndrome is classically thought to be the most common of acute kidney injury in paediatrics [10]; however, more recent data support more pre-renal and multifactorial causes [11].

Prevention of further progression and management of complications are the primary aims of treatment in children with CKD, and decisions surrounding management are complex, often due to the rarity of paediatric kidney diseases. There are few specialist centres across the country and a distinct lack of research, as is common in rarer, particularly paediatric diseases. A kidney transplant is the long-term mainstay of treatment, although there is an approximate 2-year waiting list [6], with this anecdotally being even longer in practice due to changes in deceased donor allocation preferencing. However, the true prevalence of CKD in the paediatric population is difficult to establish as the majority of data reflect end-stage kidney disease (ESKD). There are scant data available for those with mild or moderate kidney failure (CKD stages 2 to 3a, and 3b) in paediatric populations. This is something that may be key when looking at implementing another potential biomarker.

The best indicator of glomerular function and kidney function as a whole is the glomerular filtration rate (GFR). This is the rate at which substances in plasma are filtered through the glomerulus, in other words, the clearance of a substance from the blood. It is the best overall indicator of an individual’s kidney function at that time. Ideally, a marker would be an endogenous substance freely filtered at the glomerulus at a constant rate that does not undergo reabsorption, secretion, synthesis, or metabolisation by the kidney tubule and is not eliminated via any extra-renal process. There are no perfect endogenous markers with these properties that have been identified. 

There are multiple means of measuring and recording kidney function. It can be achieved directly, measured via clearance or by measuring and analysing what is filtered. Clearance is the measure of the kidney’s ability to remove a substance from the plasma and excrete it. Measuring clearance provides a preliminary estimate of the GFR. The gold standard for the measurement of GFR involves measuring plasma or urinary clearance of an exogenous marker, most commonly inulin, iothalamate, or iohexol, the measurement of which involves a continuous intravenous infusion accompanied by repeat blood and/or urine collections at carefully timed intervals. Practically time-consuming, expensive, and invasive, it is neither person- nor practitioner-friendly [12]. The commonly used clinical alternative is to calculate an estimated GFR (eGFR) based on the serum or plasma concentration of an endogenous marker such as creatinine. Creatinine is a by-product of creatinine phosphate in the muscle which is produced at a constant rate by the body and is generally cleared from the blood via the kidney, with decreased clearance resulting in increased serum creatinine [13]. 

Although there are alternative methods of estimating kidney function, we can evaluate that whilst they are less invasive, cheaper, and faster, their results may not be the most accurate. Urine albumin may be measured in 24 h urine collections or early-morning or random specimens as an albumin/creatinine ratio, with the presence of albuminuria for three or more months being indicative of chronic kidney disease [14]. These abnormal findings are often the first sign of underlying kidney disease; the higher the level of urinary protein, the poorer the prognosis. The degree of proteinuria is associated with the rate of decline in GFR and is an important consideration in the treatment and management of CKD. It has been acknowledged that haematuria is more frequent among women, whereas proteinuria more frequent among men, but there is very little mention of this impact in paediatrics. However, it is also worth noting that urinary protein or albumin levels may be increased in times of urinary tract infection, exercise, and fever [15]. 

Creatinine-calculated eGFR has historically been seen as having the closest correlation with the true GFR. It has compounding inaccuracies with its use in adults and, more so, in children. This is due to differences between ethnicities, muscle mass, age, gender, and diet. Cystatin C is an alternate endogenous marker which is not dependent on muscle mass, physical activity, or protein intake and is less influenced by age and gender. It rises faster than creatinine, aiding in earlier detection of disease [16]. This scoping review aims to explore whether cystatin C is a suitable marker for kidney function in children. It will address the potential uses as an alternative biomarker, which may be more effective in a paediatric population, possibly allowing for more accurate estimation and earlier detection of kidney function 

The aim of this review is to undertake an assessment of the available literature exploring whether cystatin C is a useful marker of kidney function in paediatric populations when compared to the more routinely used marker, creatinine.

## 2. Methodology

A search strategy focussing on studies regarding paediatrics, cystatin C, and kidney function or eGFR, plus other similar terms, was entered in Medline. The search was limited to papers in the English language and studies on human populations published after 2014, a time when cystatin C was more formally recognised [17]. Abstracts with relevance to paediatrics and cystatin C were shortlisted for full review of the text. A reverse search of identified papers was undertaken to identify further applicable papers. Two reviewers independently assessed the search yields for applicability. Where there was disagreement, a discussion was had to reach a consensus.

## 3. Calculating Creatinine-Based eGFR

In 1976, Donald W. Cockcroft and M. Henry Gault developed the first formula to predict eGFR from creatinine clearance using serum creatinine in adult males. Since then, creatinine clearance has been the most commonly used measurement of renal function. Building from this, many formulas for estimating GFR with plasma creatinine have been proposed, mainly in adult populations [18].

There has been consideration into development of paediatric creatinine equations. The most notable of these is that of Schwartz, which was formulated in the 1970s [19]. It estimates the GFR using serum creatinine, height, and an empirical constant [20] and assumes a steady state and normal body habitus [21]. More recent data suggest that this overestimates GFR [22], prompting more recent updated equations. The updated bedside Schwartz equation is most commonly used in current clinical practice in children. It is important to mention that these newly developed equations are more complicated in paediatrics due to rapid changes in weight and muscle mass. Some consider age adjustments and other biochemical markers (such as cystatin C). 

The use of creatinine itself is under great scrutiny and has been since its discovery. Shannon and Smith noted a significant difference between inulin and creatinine clearance rates in 1935 in adult populations. Based on this finding, the pair concluded that creatinine is also excreted by tubular secretion and that GFR is better measured by inulin clearance than creatinine clearance [18]. Serum creatinine levels are influenced significantly by muscle mass, diet, gender, pregnancy, and ethnicity. Due to the variable nature of creatinine production and measurement, there is an increased emphasis on the recognition of other biomarkers of kidney function that can be used to calculate eGFR. In paediatrics, serum creatinine increases gradually from one month of age because of an increase in muscle mass, and consequently, the level differs every year. Thus, reference ranges presented in broad age groups (such as 0–4, 4–7, and 7–10 years old) are clinically insufficient to use for children [23]. This also leads to further questions regarding at what point we would switch to adult equations and systems. 

## 4. Cystatin C

Cystatin C is a 13 kDa cysteine protease inhibitor, a low-molecular-weight protein produced by all nucleated cells and released into the plasma at a constant rate. Importantly, it is freely filtered at the glomerulus before being completely reabsorbed by the proximal tubules, where it is then catabolised [24]. Since its discovery in 1979, cystatin C has been recognised as a reliable marker of kidney function, with equations that are independent of sex, age, diet, ethnicity, and muscle mass. Unlike creatinine, it does not undergo tubular secretion; however, data collected from rats suggest that there is a degree of extra-renal excretion which may be as high as 15%, although this has not been confirmed in human studies [25,26].

While cystatin is considered as a reliable marker of kidney function, there are a number of factors which may affect the serum levels. Table 1 shows a summary of increasing and decreasing factors as compared to creatinine. 

Multiple studies have investigated potential new, more accurate equations for determining eGFR which include cystatin C as the leading biomarker. These are often compared with the Schwartz creatinine-based equation and a combined equation with both biochemicals considered together, often improving the accuracy of renal function assessment. A cross-sectional cohort study showed that formulae incorporating cystatin C (with or without creatinine) were more accurate for estimating GFR in a general paediatric population with a range of known clinical characteristics [27]. Work by Guido Filler et al. and Hassib Chehade et al. concluded that the Schwartz estimate showed a much larger deviation from the gold-standard derived GFR than cystatin C-based GFR estimates. It also acknowledged a substantial variability in creatinine determination, with a 20% correction factor needing to be used, though this reinforces the risk of bias in a creatinine-based formula [28]. The latter followed a similar view that new combined serum creatinine and cystatin C quadratic formulas are more accurate and should replace the combined Schwartz formula. This study did identify a further requirement for more populations with later-stage CKD (stage IV and V), and they did see an increased bias in the eGFR populations with a higher body mass index (BMI). Weight was not measured, but growth retardation and influences of muscle were identified as potential sources of inaccuracy [29].

A further comparison by M. Zapipitelli and colleagues revealed how predicted equations based on cystatin C levels are likely to provide more accurate estimates of GFR than serum creatinine-based equations, and it supported the use of cystatin C as a standard marker of GFR in children [30]. This correlates with the findings of further studies. A diagnostic accuracy study in India compared creatinine- and cystatin C-based equations in children with early CKD (eGFR validated at 60–90 mL/min/1.73 m^2^). It was determined that the cystatin C equation was more accurate and precise in the detection of early chronic kidney disease and that the addition of creatinine to the cystatin C equation did not improve its performance. A significant limiting factor in this study was the lack of non-CKD populations; it was noted that more healthy children were needed as a comparator to increase the precision in these populations [31].

## 5. Current Guidelines

Due to the absence of large, randomised trials and systematic reviews, there are currently no official guidelines in NICE regarding the use of cystatin C during diagnosis and management for both adults and children. The most recent statements did note that the use of cystatin C may reduce the number of false positive results, but may also increase the number of false negatives [32]. Taking this into account, a follow-up study researching population requirements for cystatin C testing did mention a “substantial gap between cystatin C assay requirements in primary care and national assay provision”, suggesting this was a major barrier to implementing NICE guidance [33]. On the other hand, the most recently published 2024 Kidney Disease Improving Global Outcomes (KDIGO) clinical practice guidelines recommend that, in paediatrics, an equation should be estimated using a validated GFR-estimating equation to derive GFR from serum filtration markers rather than relying on the serum filtration markers alone, though there is no mention of a specified marker in this case. The guidelines further mention that there is “currently insufficient evidence externally validated evidence to assess if combining creatinine and cystatin improves the performance of paediatric eGFR equations”. However, this is now recommended for adult practice if cystatin C is available [34]. Moreover, a National Kidney Foundation (NKF)-funded guidance study looking into equity in CKD summarised that there should be routine use of cystatin C to improve the accuracy of eGFR, though this is limited to adult populations [35]. While, in common practice, kidney function in the paediatric population is assessed using the bedside Schwartz equation, anecdotally, there is some variation in the usage of cystatin C between trusts. 

## 6. Acute Kidney Injury

In a prospective study regarding the early detection of AKI in children, it was concluded that serum cystatin C was more sensitive than serum creatinine for detecting AKI in a paediatric intensive care population. Being particularly common in this population, early detection is important for determining future management. Out of the 107 critically ill children enrolled in the study, there was no significant difference in the levels of serum creatinine in the groups with and without AKI. This was thought to be due to increased muscle wastage, loss of lean body mass, and relative malnutrition. However, there was a positive correlation between serum cystatin c and estimated creatinine clearance using the cystatin C GFR equation, with the receiver operating curve (ROC) analysis showing a significantly higher diagnostic accuracy (*p* < 0.001) with cystatin C than with estimated creatinine clearance [36].

## 7. Neonates

Studies have discussed the high variability of cystatin C in neonates, although this can be particularly challenging due to a lack of data [37]. Je-Hyan Lee et al. made a contribution to this lack of data and did conclude that reference levels of serum cystatin C should be determined according to postnatal age and postconceptional age, stating that there was a decreasing trend of cystatin C observed as post-conceptional age increased, excluding the first three postnatal days, where there was no association, with the potential for maternal factors to still contribute. More studies are required in order to confirm this. The cystatin C levels in postnatal days 4–30 were tested by correction analysis, and as a result, the cystatin C levels were negatively correlated with the gestational age at birth and post-conceptional age (*p* < 0.001), and positively correlated with postnatal age and serum creatinine (*p* < 0.001) [38]. 

A study determining neonatal kidney size and function in preterm infants, aiming to distinguish a more accurate marker of eGFR in neonates, did conclude that cystatin C equations and combined creatinine and cystatin C equations were more consistent with referenced inulin clearance studies than the creatinine equations, which underestimated GFR in neonates. Ultimately, this study concluded that cystatin C was a superior biomarker, though this was only determined after the first 48 h and first week of life. The study did acknowledge that little is known regarding the metabolism of cystatin C in pre-term infants and that more studies are needed to identify accurate measures of GFR in premature infants [39].

## 8. Ethnicity

In adults, it is thought that cystatin C does not seem to be affected by ethnicity. This has not primarily been studied in children of all ages, but a cross-sectional study with 719 children aged 12–19 in the USA determined that the mean serum cystatin C levels were significantly higher in non-Hispanic white adolescents compared to non-Hispanic black and Mexican-American adolescents. However, the magnitude of this difference was noted to be relatively small. The mean cystatin C level was 0.86 mg/L in non-Hispanic white individuals (95% CI 0.84 to 0.88) and 0.80 mg/L in non-Hispanic black individuals and Mexican-American individuals (95% CI 0.79 to 0.82). Mean serum cystatin C levels were significantly lower in non-Hispanic black (*p* = 0.003) and Mexican-American individuals (*p* = 0.001). This was compared to creatinine, which was also significantly different, where the mean serum creatinine level was 0.71 mg/dl in non-Hispanic white individuals (95% CI 0.69 to 0.73), 0.76 mg/dl in non-Hispanic black individuals (95% CI 0.74 to 0.79), and 0.67 mg/dl in Mexican-American individuals (95% CI 0.64 to 0.70), meaning that non-Hispanic black individuals had significantly higher mean serum creatinine values compared with non-Hispanic white and Mexican-American individuals (*p* = 0.004 and *p* = 0.001, respectively). Overall, this suggests that there are differences in baseline levels according to ethnicity, but this is true for both creatinine and cystatin C, and more research is needed in terms of underlying genetic factors that alter the physiologic production and/or elimination of cystatin C [40].

Another study looking into kidney size and function in a multi-ethnic population of school-aged children (age 5.6 to 7.9) investigated cystatin C and creatinine as measurements of kidney function. This cross-sectional cohort study based in in the Netherlands had a limited range of ethnicities: Dutch, Cape Verdean, Moroccan, Turkish, and Surinamese Creole, to name a few. Ethnicity was determined by the country of birth of the parents of the children, and those with congenital kidney abnormalities were excluded. It was found that there was an effect on kidney volume and function influenced by both ethnicity and body mass index, but there were no significant ethnic differences in all kidney size and function measures (all *p* < 0.05) [41]. In this trial, there was no direct comparison between biomarkers and no eGFR calculated for either, and so more studies likely need to be carried out over more varied ethnicities and much wider populations. 

## 9. Age, Height, and Gender

There is still ongoing research into calculating references ranges for ages in paediatrics. H Finney et al. describe ranges from 24-week premature infants right up the age of 17. There have been noticeable findings of peak levels of cystatin C in premature and full-term neonates, with this gradually declining until the age of one, when full adult values are reached. This has also been found to be true for creatinine, but there was a much wider variation due to a drop in the first week of life. This study also described an effect of puberty on serum creatinine levels—they reached adult levels once puberty had been reached. This clearly can be different in different genders. Overall, this study summarised that cystatin C is a better marker of GFR than creatinine in the paediatric population because it more accurately and more closely mirrors the maturation of renal function and is not confounded by other variables (e.g., increased muscle mass during growth), as is the case with creatinine [42].

Multiple studies have found that cystatin C is a more effective marker than creatinine when considering age in a paediatric population. The USA-based study mentioned above also discussed the effects of age and gender. It found that overall mean cystatin C level was higher in male over female adolescents. Overall, the mean serum cystatin C level was 0.84 mg/L, and this was significantly higher in male compared with female individuals (0.89 versus 0.79 mg/L, *p* < 0.001). This equated to around 12.7% higher serum levels in males. This was repeated with serum creatinine levels, with the mean serum creatinine levels being 0.72 mg/dL and 0.77 mg/L in males versus 0.66 mg/dL in females (*p* < 0.001), i.e., 16.7% higher.

Cystatin C levels were found to peak at 12 years in females, but they were highest in males at age 14, with both levels decreasing after this. This may indicate that other factors are present, such as hormonal variations or other influences regarding the development of puberty that may interfere with the cystatin C assay/production. This is something that may require further investigation. Mean creatinine levels, in comparison, sharply increased with age for both male and female individuals between ages 12 and 19. In unadjusted analysis, each 1-year increase in age was associated with a 0.029 mg/dL increase in serum creatinine, and age accounted for approximately 19.7% of the variation in serum creatinine levels. This is similar to the effect of gender on creatinine. Overall, this indicates that age is less likely to affect serum cystatin C levels as compared to creatinine. 

However, it is worth noting that this study did not directly measure the effect on eGFR. However, in a combined comparison of the effects of age, gender, and race on cystatin C levels with creatinine, it was found that gender and race have comparable magnitudes of effect on cystatin C and creatinine, whereas age has a much smaller effect on cystatin C than on creatinine [40].

The aforementioned study only looked at ages 12–19; another study by Fischbach et al. looked at the impact of age on cystatin C levels in 98 healthy children, with 51 of those being under 18 months of age. It was discovered that infants under 18 months had higher mean serum cystatin C of 0.94 mg/L (±0.24 mg/L) than older children (0.65 ± 0.19 mg/L). Under this age, there was a negative correlation between age and cystatin c (r^2^ = 0.631, *p* < 0.01). In summary, the results indicated that the mean serum cystatin C was higher in infancy than in older children, but that there was an age cut-off of 1 year, potentially reflecting kidney maturation. This was a limitation of the study, as there was no accurate assessment of the age cut-off at 18 and 36 months, on top of the lower number of participants [43].

The only study that truly compared the full effect of age (and height) on GFR was the study of 184 children 0.24–17.96 years old in Germany. In this, serum cystatin C and serum creatinine were measured against inulin clearance. It was concluded that cystatin C (r = 0.88) correlated better with inulin clearance than creatinine (r = 0.72). It was identified that height was a covariate for creatinine, but there were no covariates for cystatin C. Female gender and dystrophy were associated with an overestimation of GFR from serum creatinine and height, but the inulin-derived GFR was in line with the cystatin C-derived eGFR. Overall, it was calculated that a cut-off cystatin C concentration of 1.39 mg/L had a 90% sensitivity and 86% specificity for detecting an abnormal GFR, possibly indicating great potential for a future biomarker [44].

## 10. Urinary Tract Malformations

Urinary tract malformations are a significant paediatric issue, and the impact on cystatin C is generally undecided. One prospective study looked at 72 healthy children with urinary tract malformations to determine the potential impact of cystatin C versus creatinine. It was revealed that cystatin C had a higher correlation (r = 0.62, *p* < 0.001) with the gold-standard ⁹⁹ᵐTc DTPA-derived GFR than creatinine (r = 0.51, *p* < 0.001). This was most marked in those with mild renal impairment and uropathy. Mean differences for the gold standard and cystatin C were −2.6% ± 46.7% and −73.4% ± 53.6% for creatinine. Cystatin C performed better in the 0-to-6-month-old category and in patients 12 months and older. Cystatin C was proven to be the superior marker in this case for estimating GFR in children younger than 3 years with urinary tract malformations [45].

## 11. Research Challenges

The investigation of kidney function in children and adolescents is more complicated than in adults—there are the issues of continuing kidney development, fluctuating muscle mass, and the interference of haemoglobin and bilirubin that can take place in neonates. Furthermore, inborn errors of metabolism are more prevalent in paediatric populations. A low-protein diet may be more necessary, and many can also cause progressive kidney impairment. A marker such as cystatin C would be much more advantageous than creatinine in these situations. Moreover, there are currently insufficient externally validated data to assess whether combining creatinine and cystatin C improves the performance of paediatric eGFR equations.

In children, there is a paucity of evidence of other, non-creatinine endogenous markers of eGFR. Studies have tended to be of a smaller size, perhaps due to more rigorous ethical regulations [46]. Nevertheless, while there are many more papers regarding the use of cystatin C in adults, it is not well documented or understood in terms of paediatrics, and so this literature review discusses the potential for its use as a biochemical marker of kidney function in children.

Further limitations of cystatin C implementation are its cost and accessibility. Cystatin C and creatinine are both run on standard automated lab analysers with low labour costs. However, the cystatin C reagent costs around USD 4 per test compared to USD 0.20 for creatinine, and this is thought to decrease with further usage. However, this is comparable to the expenses of other common tests which nephrologists will frequently order, such as parathyroid hormone (USD 3.25), 25-hydroxyvitamin D (USD 7.00), and c-reactive protein (USD 2.75) [47].

The preferred assay for large-scale, commercially available auto-analyser measurements of creatinine is the enzymatic method. This is in preference to the alkaline picrate measure. In comparison, for measurement of cystatin C, there are two primary methods utilising particle-enhanced immunoassay technology. These are particle-enhanced turbidimetric immunoassay (PETIA) and particle-enhanced nephelometric immunoassay (PENIA). These provide fast turnaround, higher precision, and are less likely to be affected by interfering substances when compared to creatinine [16,48].

**Figure 1:**
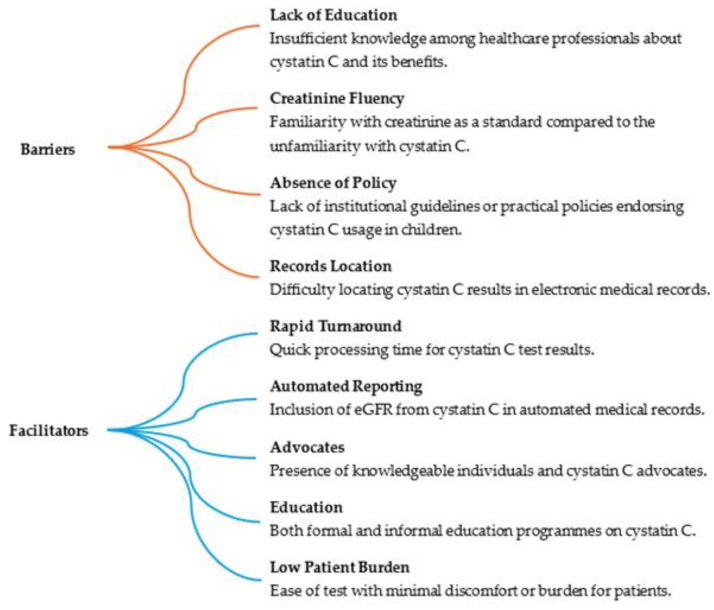
Factors influencing cystatin C utilisation [49].

Awareness and clinician confidence is another barrier to further usage. In a qualitative study by the Mayo clinic of 15 clinicians in various disciplines with varying knowledge of cystatin C and comfort with its use, a number of factors were identified affecting its usage. The results are shown in Figure 1 [49].

In a retrospective study of primary care laboratories in Oxfordshire which determined the population requirement for cystatin C assay testing compared to national availability, it was found there was a substantial difference, with only a small minority of clinical chemistry laboratories providing testing. This proves to be one of the greatest barriers to the implication of cystatin C on a more national level [33].

## 12. Conclusions

Our study highlights the complexities of diagnosing CKD in children, particularly the need for age-appropriate criteria due to varying GFR values. It compares current kidney function biomarkers, noting creatinine’s limitations due to factors such as muscle mass, diet, and ethnicity. This study emphasizes cystatin C as a potentially more reliable biomarker which is less influenced by these variables for better assessment and early detection of kidney dysfunction in paediatric patients. Our findings suggest that incorporating cystatin C into routine practice could improve CKD diagnosis accuracy, although current infrastructure gaps need to be addressed. The current data suggest that cystatin C is a more reliable and consistent measure of kidney function in paediatric populations. It was better in the earlier detection of AKI in a paediatric intensive care population and more accurate than creatinine in detecting renal function in neonates. Overall, cystatin C is a more precise marker in those with urinary tract malformations, and it is affected by fewer variables in terms of gender and age. 

More research and studies in this population are clearly needed, as only smaller studies have been published. There is growing evidence of the effectiveness of cystatin C in a calculated equation, but no guidelines take cystatin C into account currently. A significant limitation of cystatin C is the wide gap between requirements for mass usage and the actual provision of readily available assays. Large-scale, high-quality studies are needed in order to develop guideline recommendations. 

## Figures and Tables

**Table 1:** Summary of influencing factors of cystatin C and creatinine [16].

Cystatin C	Creatinine
Increasing Factors	
Steroid treatment Current cigarette smoking Chronic inflammation Obesity Hyperthyroidism	Black ethnicity Increased muscle mass High-protein diet and supplements Rhabdomyolysis—intense exercise/muscle damage Testosterone Drugs—cimetidine, trimethoprim
Decreasing Factors	
Hypothyroidism	Increasing age Female gender Asian ethnicity Inflammation Neuromuscular illness or amputation Malnutrition hyperthyroidism
